# Knowledge, attitude, and practice toward COVID-19 transmission, prevention, and self-quarantine management among public servants in selected locations of the Sidama region, Southern Ethiopia: a multicenter cross-sectional study

**DOI:** 10.3389/fpubh.2023.1170317

**Published:** 2023-06-27

**Authors:** Yacob Abraham Borie, Tomas Yeheyis, Bedilu Deribe, Mohammed Ayalew, Yared Reta, Dawit Hoyiso, Wegene Jembere

**Affiliations:** School of Nursing, College of Medicine and Health Sciences, Hawassa University, Hawassa, Ethiopia

**Keywords:** COVID-19, awareness, perspective, implementation, lockdown, health, quarantine, civil servant

## Abstract

**Background:**

The COVID-19 epidemic has put an enormous strain on the world’s healthcare systems, lifestyles, and quality of life. Ethiopia attempted to meet the myriad needs of its people due to the COVID-19 epidemic and the government has demonstrated a strong commitment in order to lessen the epidemic’s impact on the populace. Despite this fact, the population’s compliance with measures was not as needed.

**Objectives:**

To assess knowledge, attitude, and practice regarding COVID-19 transmission, prevention, and self-quarantine management among public employees in selected locations of the Sidama Region, Southern Ethiopia, in 2020.

**Methods:**

An institution-based cross-sectional study was conducted from 01 October to 30 October 2020, among 399 public servants in selected locations of the Sidama Region, Sothern Ethiopia. One-stage cluster sampling was used to randomly select 16 public service sector offices from the total 32 sector offices in the selected locations of the region. Simple random sampling was employed to select respondents following equal distribution of the samples to 16 sector offices. Data were collected using an adapted self-administered questionnaire. Data entered using EpiData version 3.1 and SPSS version 24 were used for statistical analysis. Descriptive statistics was used to compute frequencies, percentages, and means for independent and dependent variables.

**Result:**

Overall, 42.36% of respondents had good knowledge of COVID-19, while the remaining 57.64% had poor knowledge. The percentage of favorable attitudes toward COVID-19 prevention and control were 65.2, 54.4% of respondents had a good level of practice of COVID-19 preventive and control measures, and 52.4% of the respondents had a good level of knowledge regarding self-quarantine management.

**Conclusion:**

The level of knowledge, attitude, practice, and self-quarantine management in the area is insufficient for preventing and controlling the disease. Evidence-based awareness creation and law enforcement in the study areas and surroundings, with an emphasis on infection prevention and control (IPC) in the public sector and other public gathering areas, is recommended.

## Introduction

Worldwide healthcare systems, as well as people’s lifestyles and quality of life, are all being strained by the COVID-19 epidemic. Ethiopia attempted to meet the myriad needs of its people as the COVD-19 epidemic was approaching. The government demonstrated a strong commitment to lessening the epidemic’s impact on the populace (1).

Fever, headache, cough, and shortness of breath are some of the non-specific symptoms that can occur. Acute respiratory distress syndrome, the most severe of the clinical manifestations that necessitate mechanical ventilator support to preserve life, can develop in COVID-19 patients with underlying medical conditions (2). According to reports, the majority of deaths happened in adults over the age of 50, followed by small children (3).

By March 2020, the WHO had detected community transmission in some African countries, including Ethiopia, and the risk of coronavirus spreading was due in part to deep challenges in practicing social distancing and frequent hand washing in settings with high population densities and lack of running water, as well as COVID-19’s non-specific symptoms, which make it difficult to distinguish from endemic illnesses like malaria and influenza (4).

In May 2020, Africa had 87,925 confirmed COVID-19 cases spread across 55 nations. The biggest number of cases were in South Africa, where there were 16,433 instances (or 18.7% of the total), followed by Egypt with 12,229 cases (or 13.9%), Algeria with 7,019 cases (or 8.0%), Morocco with 6,952 cases (7.9%), Nigeria with 5,959 cases (6.8%), and Ghana with 5,735 cases (or 6.5%) (5).

The lack of publicly shared knowledge has been a challenge not only in the face of increased worries about fake news but also in the face of widespread distrust of public institutions that promote individual behavior norms and coordinate communal cooperation during epidemics (6, 7).

To stop the transmission of coronavirus, people must adopt personal hygiene and public health behaviors, including hand washing and social distancing, although this will be difficult in many cities and rural areas in poor countries (8). Ethiopia’s government instituted strong control measures after confirming its first incidence of COVID-19 on March 12th, 2020, and the response was increasingly forceful as the days passed. Social distancing, continual cleanliness, the use of face shields, limiting public vehicle traffic, shutting down public facilities that did not dispense vital supplies, and limiting traffic hours to specified daily hours were all enforced (1).

Even though public preventive measures were mandated, adherence to each of them was somewhat weak among the population. Non-compliance and, to some extent, apathy on the part of certain human groups about these regulations was concerning. Cross-sectional studies identified this phenomenon as an attitudinal problem attributable to the population. One of the first and most current studies on coronavirus attitudes and knowledge, conducted in Hubei, found that opinions toward government actions to manage the epidemic were strongly linked to awareness about COVID-19. The authors explained that the more information and education people have, the more positive attitudes they have concerning COVID-19 prevention efforts (9, 10).

A major obstacle to controlling the pandemic was the lack of understanding and biased attitudes surrounding the prevention of COVID-19 transmission. Poor understanding, attitudes, and practice had a big influence on the fight against the coronavirus in nations like Ethiopia, in addition to the nation’s limited testing capabilities and management. These findings would help policy makers, the academic community, and the partners working on COVID-19 have better understanding before taking action.

We created a poll to study this topic due to the scarcity of previous studies on epidemics, knowledge, and attitudes in our nation. This study aimed to see how much people know about COVID-19’s most prevalent symptoms, transmission pathways, and severity, specifically to investigate civil servants’ perceptions of the disease’s threat or severity, as well as the behaviors developed in response to it in the Sidama Region.

## Methods and materials

### Study area

The Sidama Region is the newly established regional state of the Southern Nations, Nationalities, and People’s Region (SNNPR). The study was conducted in the city of Hawassa, and the towns of Yirgalem and Leku. Hawassa is the capital of the region, containing eight sub-cities and 32 kebeles (20 urban and 12 rural). Yirgalem and Leku towns are located 33 km and 10 km away from Hawassa, respectively.

### Study design and period

An institution-based cross-sectional study was conducted from 01 October to 30 October 2020, among 399 public servants in the selected locations of the Sidama Region, Sothern Ethiopia. Public servants in the chosen locations used an interviewer-administrated questionnaire.

### Sample size determination

The required sample size was determined using a single population formula where the proportion of public servants with knowledge, attitude, and practice (KAP) toward COVID-19 transmission prevention and self-quarantine management in the study area was unknown. Hence, a 50% prevalence (*p* = 0.5) was taken to calculate the sample size. The chosen margin of error was 5% with a 95% confidence interval, and, considering the no response rate was 10%, the final sample size yielded was 422.

### Source population

All public personnel in specific locations’ administrations were used as the source population.

### Study population

All public officers working in specified public sectors who were accessible during the study period made up the study population.

### Eligibility criteria

All public servants in selected public sectors and who were working in the office during data collection time. Public servants who graduated in health and health-related fields and those working as members of the COVID-19 prevention task force were excluded from the study.

### Sampling procedure

The respondents were chosen using one-stage cluster sampling. The 32 sector offices offer a variety of services to the public. Of these, 16 public service sector offices were chosen at random using a lottery system, accounting for half of all the public service offices. Then, the sample size was selected and divided evenly among the 16 sector offices. Finally, using a simple random sampling procedure, the assigned number of samples were collected from sector offices. Before using the basic random sampling technique, a sampling frame was created for each selected sector based on the alphabetical order of the selected public service offices.

### Data processing and analysis

EpiData version 3.1 was used to enter data, which were then exported to SPSS version 24 for statistical analysis. For independent and dependent variables, descriptive statistical analysis was used to compute the frequencies, percentages, and means.

### Data quality assurance and tool

A standardized and pre-tested questionnaire was used to ensure data quality. A pre-test was done on 5% of the sample size. The validity of the questionnaire was conducted using Pearson’s product moment correlations by correlating each questionnaire item with the total score using SPSS. The total linear correlation coefficient (xry) of 0.712 was greater than the r table product moment for each item assessing KAP. The reliability of the variables was checked using Cronbach’s alpha before data collection. The internal consistency of Cronbach’s alpha for items assessing knowledge, attitude, and practice was 0.82, 0.8, and 0.78, respectively.

Before data collection, data collectors and supervisors were given two days of training. COVID-19 prevention and transmission were the topics of the training. During the data collection, supervisors checked daily for consistency and completeness of the data, and, at the end of the day, all investigators collected all completed questionnaires from the data collectors and checked for completeness and consistency.

### Operational definitions

*Knowledgeable*: participants who scored the mean value or above for the provided knowledge-based questions.

*Poor knowledge*: participants who scored below the mean value for the provided knowledge-based questions.

*Favorable attitude*: participants who scored above the mean value for the provided attitude-related question.

*Unfavorable attitude*: participants who scored below the mean value for the provided attitude-related questions.

*Good practice*: participants who scored greater than the mean value for the provided practice-based questions.

*Poor practice*: participants who scored below the mean value for the provided practice-based questions.

### Ethical consideration

The Hawassa University College of Medicine and Health Sciences’ health ethical review board provided ethical approval and clearance. The goal of the study was explained to the participants, and they gave their agreement. The right to drop out of the study at any time was guaranteed. To maintain participant confidentiality, coding was utilized to remove names and other personal identifiers from respondents throughout the study procedure.

## Results

### Socio-demographic characteristics of the respondents

A total of 399 public servants responded to the study, yielding a response rate of 94.5%. Of the total respondents in the study, more than half (53.6%) were male, 64.9% were married, 76.7% had a degree or above in their educational status, and the mean age of the respondents was 32.31 years with a standard deviation of 6.77 (Table 1).

**Table 1:** Sociodemographic characteristics of study participants among selected public servants in the Sidama Region, 2020.

Characteristics	Category	Frequency	Percent
Sex	Male	214	53.6
	Female	185	46.4
Age (years), categorized	20–25	62	15.5
	26–30	142	35.6
	31–40	79	19.8
	41–56	116	29.1
Religion	Protestant	261	65.4
	Orthodox	113	28.3
	Muslim	7	1.8
	Catholic	10	2.5
	Others, specify	8	2.0
Marital Status	Single	131	32.8
	Married	259	64.9
	Widowed	3	0.8
	Separated	6	1.5
Education status	Primary to high school	10	2.5
	Certificate	7	1.8
	Diploma	76	19.0
	Degree and above	306	76.7
Residence	Yirgalem	88	22.1
	Leku	108	27.1
	Hawassa	203	50.9

### Source of information on COVID-19

Mass media was the main source of information for the respondents in this study (76.2%) and more than half of the respondents (55.6%) shared the information they found with friends, family, and society (Table 2).

**Table 2:** Information source and dissemination of COVID-19 infection prevention and transmission among selected public servants in the Sidama Region, 2020.

Characteristics	Category	Frequency	Percent
Source of information	Mass media	304	76.2
	Social media	37	9.3
	Friends	11	2.8
	Families	18	4.5
	Journals	29	7.3
Sharing of information	Family	84	21.1
	Friends	30	7.5
	Friends and family	63	15.8
	Friends, family, and society	222	55.6

### Knowledge of COVID-19 infection prevention and transmission

Overall, 42.5% of respondents had good knowledge of COVID-19 infection prevention methods and modes of transmission with a median (IQR) of 13 (12–14) (Table 3).

**Table 3:** Knowledge of COVID-19 infection prevention and transmission among the corresponding locations of the selected public servants in the Sidama Region, Ethiopia, 2020.

Town	Knowledge		Total
	Poor	Good	
Yirgalem	45 (51.13%)	43 (48.3%)	88
Leku	62 (57.4%)	46 (42.6)	108
Hawassa	123 (60.6)	80 (39.4%)	203
All	230 (57.64)	169 (42.35)	399

### Attitude toward COVID-19 infection prevention

Overall, 65.2% of respondents in the three locations had a favorable attitude, and 34.8% had an unfavorable attitude toward COVID-19 infection prevention and transmission (Table 4).

**Table 4:** Attitude toward COVID-19 infection prevention and transmission among the corresponding locations of the selected public servants in the Sidama Region, Ethiopia, 2020.

Town	Attitude		Total
	Poor	Good	
Yirgalem	34 (38.6%)	54 (61.4%)	88
Leku	35 (32.4%)	73 (67.6%)	108
Hawassa	70 (34.5%)	133 (65.5%)	203
All	139 (34.8%)	260 (65.2%)	399

### Practice toward COVID-19 infection prevention and transmission

More than half of the respondents in this study, 54.4%, had a good level of practicing COVID-19 infection prevention and control measures, whereas the rest, 45.6%, recorded a poor level of practicing infection prevention and control measures (Table 5).

**Table 5:** Practice toward COVID-19 infection prevention and transmission among the corresponding locations of the selected public servants in the Sidama Region, Ethiopia, 2020.

Town	Practice		Total
	Poor	Good	
Yirgalem	29 (33%)	59 (67%)	88
Leku	92 (85.2%)	16 (14.8%)	108
Hawassa	61 (30.0%)	142 (70.0%)	203
All	182 (45.6%)	217 (54.4%)	399

### Self-quarantine management toward COVID-19 prevention and control

**Figure 1:**
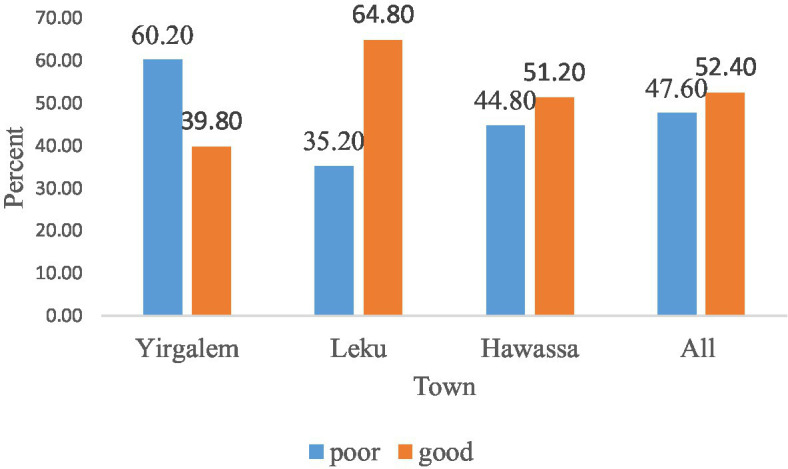
Knowledge of self-quarantine management for COVID-19 infection prevention and transmission among the corresponding locations of selected public servants in the Sidama Region, Ethiopia.

Half of all participants in the study, 52.4%, had a good knowledge of self-quarantine management protocol for COVID-19. The rest had poor knowledge of staying at home in a private room if exposed or having a contact history (Figure 1).

## Discussion

Our research aimed to determine public servants’ attitudes toward COVID-19 in Hawassa city, Leku, and Yirgalem.

The investigation looked into where COVID-19 information came from. The bulk of the research participants (76.2%) got their knowledge from the mass media and then followed it up with social media. This suggests that the media plays a crucial role in disseminating vital public health information. A study from Addis Ababa supports our findings (11) and, according to a study in Iran (12), COVID-19 information was most commonly obtained from the media and short message services (SMS). This backed up a study from the Philippines that showed traditional media outlets like television and radio were the primary sources of knowledge about the virus (13). In contrast, a recent Ethiopian study found that social media was the primary source of information (14).

In our study, 42.35% of participants had a strong understanding of how to prevent COVID-19 infection and transmission. In the study area, however, there was some variation between cities. As a result of this finding, public officials in various healthcare sectors will have varying levels of access to COVID-19 information. This finding is in line with a study undertaken in the Tigray Region (15).

Participants in this study had a better degree of knowledge than those in the Addis Ababa study (11). In Addis Ababa, however, the proportion of high-risk groups with good knowledge was 52%, which is greater than the current study (16).

This discrepancy may be attributable to differences in the study populations. Furthermore, persons who are at high risk may have a stronger desire to learn about COVID-19 than others. The current study’s findings differ from those of Chinese studies (17) and one conducted in Arba Minch (18). The proportion of participants with good knowledge in the current study was similar to that found in an adult population study in the Sidama Region (19).

The majority of respondents (65.2%) in this study had a favorable opinion toward COVID-19 infection prevention and control. This result is lower than that of a Nigerian study (79.5%) and also in Uganda, where 72.4% had a good attitude (20). Differences in the study site, demographics, and tools used could explain the disparity.

These results (61%) are very similar to those of the Gondar study (21). In our study, 57.6% of people believed that COVID-19 infection is spiritual or that it is caused by sin as a punishment from God. One of the primary obstacles in preventing and controlling COVID-19 could be a pessimistic attitude. This idea is similar to one expressed in a Ghanaian report (22).

Furthermore, traditional medicine is trusted by 41.6% of the survey participants as a remedy for the ailment. This mindset may lead to neglect and a deterioration of the healthcare delivery system.

According to the results of this study, 217 (54.4%) of the participants had good practice regarding COVID-19 and its prevention. This suggests that a large number of government employees were not following the health sector’s and government’s infection prevention and control messages. Our findings are higher than those of a study conducted in Debre Tabor. However, regarding hand washing practice, in our study, 55.9% reported proper hand washing as a means of COVID-19 prevention. This finding is lower than the study in Debre Tabor, which was 67.2% (23). According to a study conducted in Arba Minch, 33.3% of people wore a mask while going out (18), whereas in our study, 64.2% reported they wore a mask while going out. This difference may be due to variations in law enforcement in the study areas and differences in the study population. Our study area’s COVID-19 prevention practice was lower than that of a study done in China (17).

According to the assessment, 52.4% of self-quarantine management against COVID-19 transmission was good, while 47.6% was bad. The majority of participants (74.2%) stated that they would stay at home in a secluded room and avoid social events to decrease COVID-19 transmission. This figure is lower than the 95.9% figure reported in a Saudi study (24). Even though the majority of survey participants (78–81%) said they would get tested voluntarily if they had a contact history, were exposed to COVID-19, or experienced COVID-19 symptoms, a significant number of participants still had reduced regard for COVID-19 transmission control. Furthermore, 27.6% said they would not refrain from sharing equipment during self-quarantine and 15.8% of respondents stated that they lacked knowledge about self-quarantine management.

## Conclusion

This study indicates that the current level of knowledge, attitude, practice, and self-quarantine management is insufficient for preventing and controlling the disease and that evidence-based awareness creation and law enforcement in the study areas and surroundings, particularly in the public sector and other public gathering areas, is required. The results imply that poor hygiene practice would perpetuate the spread of the infection in the county and create a barrier for early infection mitigation. In both governmental and non-governmental sectors, as well as public events, wearing a mask and maintaining a physical distance should be frequently practiced.

## Limitations

- The associated factors have not been examined; only descriptive components have been presented.
- The results may not adequately explain the current COVID-19 situation in Ethiopia due to the timing of the report.
- Practice was not assessed using observational methods.

## Data availability statement

The original contributions presented in the study are included in the article/Supplementary material, further inquiries can be directed to the corresponding author.

## Ethics statement

The study protocol was ethically approved by the Institutional Review Board (IRB) of Hawassa University, College of Medicine and Health Sciences. In addition, the study was conducted following the Declaration of Helsinki. Moreover, the confidentiality of information was guaranteed by using code numbers rather than personal identifiers and by keeping the data locked. The patients/participants provided their written informed consent to participate in this study.

## Author contributions

YB, BD, MA, and YR conceived and designed the study. DH, WJ, and TY assisted with the design conception, advising, and critical reviewing of the research report. YB and TY prepared the manuscript. All authors have read and approved this manuscript.

## Conflict of interest

The authors declare that the research was conducted in the absence of any commercial or financial relationships that could be construed as a potential conflict of interest.

## Publisher’s note

All claims expressed in this article are solely those of the authors and do not necessarily represent those of their affiliated organizations, or those of the publisher, the editors and the reviewers. Any product that may be evaluated in this article, or claim that may be made by its manufacturer, is not guaranteed or endorsed by the publisher.
