# Supplementary material for: Knowledge, attitude, and practice toward COVID-19 transmission, prevention, and self-quarantine management among public servants in selected locations of the Sidama region, Southern Ethiopia: a multicenter cross-sectional study
Source: Front Public Health. 2023 Jun 27;11:1170317. doi: 10.3389/fpubh.2023.1170317 (PMC10335795; doi:10.3389/fpubh.2023.1170317)
Supplement: Supplementary file 1 [file Data_Sheet_1.pdf]

**Part II knowledge about COVID19 among Hawassa City public servants ,2020**

| s.no | Questions | Yes | No | I don't know |
|---|---|---|---|---|
| K1 | Eating or contacting wild animals would result in the infection by the COVID-19 virus. | | | |
| K2 | Persons with COVID-19 cannot infect the virus to others when a fever is not present. | | | |
| K3 | The COVID-19 infection spreads via respiratory droplets of infected individuals. | | | |
| K4 | Smokers and tobacco users are at higher risk of COVID-19 infection | | | |
| K5 | Isolation and treatment of people who are infected with the COVID-19 virus are effective ways to reduce the spread of the virus. | | | |
| K6 | Ordinary residents are advised to wear face-masks to prevent COVID-19 infection. | | | |
| K7 | Ordinary residents are advised to wear glove to prevent COVID-19 infection. | | | |
| K8 | It is not necessary for children and young adults to take measures to prevent the infection by the COVID-19 virus. | | | |
| K9 | Individuals should avoid going to crowded places such as train stations and avoid taking public transportations. | | | |
| K10 | People who have contact with someone infected with the COVID-19 virus should be immediately isolated in a proper place. In general, the observation period is 14 days. | | | |
| K11 | Wash your hands often with soap and water is advisable especially after you have been in a public place, or after blowing your nose, coughing, or sneezing. | | | |
| K12 | Put distance between yourself and other people is important if COVID-19 is spreading in your community. | | | |
| K13 | To prevent the infection by COVID-19, you should avoid touching your eyes, nose, and mouth with unwashed hands. | | | |
| K14 | Not all persons infected with COVID-19 virus will develop severe complications. Only those who are elderly, have chronic illnesses, and obese are more likely to be severe complication. | | | |

| | | | | |
|---|---|---|---|---|
| **K15** | Currently, there is no effective treatment for COVID-19 infection, but early symptomatic and supportive treatment can help most patients to recover from the infection. | | | |

**Part III. Attitude towards COVI19prevention, transmission among Hawassa City Public servants ,2020**

| s.no | Questions | Yes always ,most of time | No , sometime, rarely |
|---|---|---|---|
| **A1** | Do you agree that COVID-19 will finally be successfully controlled? | | |
| **A2** | Do you have confidence that world leaders (WHO) can win the battle against the COVID-19 virus? | | |
| **A3** | Do you think that the cause of Covid-19 is spiritual/ is it happened because of our sin? | | |
| **A4** | Didn't you generally oppose the wearing of face mask? | | |
| **A5** | Information about the coronavirus seems to spread as fast as the virus itself. So, are you negatively affected by the news you hear about COVID-19 infection? | | |
| **A6** | Do you think that infected person can cure from the COVID-19 infection with traditional medicines? | | |

**Part IV. Practice towards preventing COVID19 among Hawassa city public servants ,2020**

| | Questions | Yes always , most of time | No , sometime, /rarely |
|---|---|---|---|
| P1 | In recent days, have you restricted going to any crowded place? | | |
| **P2** | In recent days, have you worn a mask when leaving home? | | |
| **P3** | Have you wash your hands often with soap and water for at least 20 seconds especially after you have been in a public place, or after blowing your nose, coughing, or sneezing? | | |
| **P4** | If soap and water are not readily available, are you applying hand sanitizer that contains at least 60% alcohol? | | |
| **P5** | Do you use sterilizers before and after touching inanimate object like money, after being in contact with ATM etc.? | | |
| **P6** | Do you apply if the government announce stay-at-home order? | | |
| **P7** | Didn't you touch your nose and mouth frequently without washing your hand? | | |

**Part V self-quarantine management for preventing and transmission of COVID 19**

1. Have you heard of self-quarantine in case of COVID19?

I. yes II. No

2. If yes, what do you understand by self -quarantine? You can select more than one

I. it is isolating one -self from others yes no

II. Living alone in private home yes no

Iv. Avoiding sharing equipment with others yes no

V. avoiding social life yes no

VI. Avoiding physical distance from others yes no

VII. Avoiding public gathering

2. If you had contact with suspected or confirmed case of COVID 19, How would you prevent transmission for others

I. staying at private home for 10 to 14 days yes no

II. Avoiding physical distance at least 2 meters apart with roommates yes no

3. If you had contact with suspected or confirmed case of COVID19, would you voluntarily go for test?

I. yes II. no

4. If you had symptoms of COVID19, what would you do?

1. I would go for test voluntarily yes no

5.Do you think that early symptomatic and supportive treatment can help most patients to recover from COVID19 infection

I. yes II. no
